# Hypogonadism in Exercising Males: Dysfunction or Adaptive-Regulatory Adjustment?

**DOI:** 10.3389/fendo.2020.00011

**Published:** 2020-01-31

**Authors:** Anthony C. Hackney

**Affiliations:** Department of Exercise and Sport Science, Department of Nutrition, Gilling's School of Global Public Health, University of North Carolina, Chapel Hill, NC, United States

**Keywords:** testosterone, sport, androgens, athletes, impairment, sex

## Abstract

For decades researchers have reported men who engaged in intensive exercise training can develop low resting testosterone levels, alterations in their hypothalamic-pituitary-gonadal (HPG) axis, and display hypogonadism. Recently there is renewed interest in this topic since the International Olympic Committee (IOC) Medical Commission coined the term “Relative Energy Deficiency in Sports” (RED-S) as clinical terminology to address both the female-male occurrences of reproductive system health disruptions associated with exercise. This IOC Commission action attempted to move beyond the sex-specific terminology of the “Female Athlete Triad” (Triad) and heighten awareness/realization that some athletic men do have reproductive related physiologic disturbances such as lowered sex hormone levels, HPG regulatory axis alterations, and low bone mineral density similar to Triad women. There are elements in the development and symptomology of exercise-related male hypogonadism that mirror closely that of women experiencing the Triad/RED-S, but evidence also exists that dissimilarities exist between the sexes on this issue. Our research group postulates that the inconsistency and differences in the male findings in relation to women with Triad/RED-S are not just due to sex dimorphism, but that there are varying forms of exercise-related reproductive disruptions existing in athletic men resulting in them displaying a relative hypogonadism condition. Specifically, such conditions in men may derive acutely and be associated with low energy availability (Triad/RED-S) or excessive training load (overtraining) and appear transient in nature, and resolve with appropriate clinical interventions. However, manifestations of a more chronic based hypogonadism that persists on a more permanent basis (years) exist and is termed the “Exercise Hypogonadal Male Condition.” This article presents an up-to-date overview of the various types of acute and chronic relative hypogonadism found in athletic, exercising men and proposes mechanistic models of how these various forms of exercise relative hypogonadism develop.

## Introduction

Many national and international organizations have touted the health benefits of being physically activity and engaging in exercise training (1, 2). Research evidence is overwhelmingly supportive that an active lifestyle leads to improved quality and quantity of life for individuals (3, 4). For this reason, many public health professionals are promoting and encouraging the populations within their respective countries to adopt behaviors that incorporate more physical activity into their daily living. To this end, the concept of using physical activity and exercise training as a preventative healthcare adjunctive therapy has become a popular contemporary theme. Furthermore, this is sound medical policy as preventative steps in promoting improved health are typically far most cost-effective and successful than interventional alternatives (5).

However, exercise is not a panacea for all human afflictions and ills and in and of itself can induce health complications (*N.B.*, for convenience in this paper the term *exercise* is used to refer to both physical activity and exercise). Most healthy individuals recognize that by doing more exercise the risk for musculoskeletal injury increases; but, what most do not know, however, is other complications can present themselves with exercise. In particular many in the general public are unaware of how increasing levels of exercise can precipitate endocrine dysfunction by promoting changes in circulating hormone levels (the term *dysfunction* and *disorder* are used interchangeably by researchers, this article uses the term dysfunction). Although it is important to note, such occurrences are primarily associated with individuals who perform exercise at levels beyond the recommendations for health and physical fitness improvement (6). That is, specifically, men and women who are conducting exercise training at levels to allow themselves to be highly competitive in sporting events are more at risk.

Perhaps the most notable endocrine dysfunction linked to exercise training is that which involves disrupts in a woman's reproductive system leading to the development of secondary amenorrhea—what was originally referred to as “athletic amenorrhea.” This occurrence is now recognized as part of the consequences of the medical condition known as the Female Athletic Triad (Triad) which is associated with increased risk for infertility, bone mineral loss, potentially disordered eating behaviors as well as reduced reproductive hormone levels (7). In the 1970's medical researchers began to understand that exercise training could have these negative consequences in women. Landmark research studies by scientists such as Drs. Anne Loucks, Constance Lebrun, Naama Constantini, Michelle Warren, and the late Barbara Drinkwater, to name just a few, laid the groundwork for this important medical finding.

Less familiar to the public is the influence of exercise training on the reproductive endocrinology of men. For many years researchers assumed the male reproductive system was robust enough to tolerate the stress of demanding levels of exercise training and was thus unaffected. Today we know that is not the case and in fact, there are many similarities in the aspects of the reproductive dysfunctions that develop in women and men. The degree and scope of the research on men are far more limited than that in women; and, perhaps rightly so due to the prevalence and severity of the health consequences found in women with the Triad.

The research addressing reproductive dysfunctions in men began later than that involving women and was pursued by a very limited number of researchers for many years. Today the number of researchers and studies addressing men on this issue has grown dramatically; and, now more attention is being focused than ever before on the negative reproductive health consequences suffered by men engaged in exercise training.

The growth and expansion of interest in the male reproductive system as an exercise research topic is long overdue and it is exciting to see many new researchers now pursuing this line of work. But, the rapid expansion of interests in this topic has led to some misconceptions and misunderstandings by the general public as well as some in the research community concerning male endocrinology and the reproductive hormonal anomalies associated with exercise training. These occurrences have developed for several reasons: (1) misinformation or overly simplified information presented on internet exercise websites; (2) lack of general familiarity with the nearly three-plus decades of prior research already done on men and reproductive dysfunction; (3) faulty assumptions that all exercise reproductive dysfunction in men are of one causation—i.e., the “one size fits all” explanation, and (4) the application of finding on reproductive dysfunction in women being directly translated and applied to men.

This review article intends to clarify some of these misconceptions and misunderstandings and provide historical background and physiological overview of reproductive dysfunctions found in men engaged in exercise training—specifically, focusing on the development of exercise relative hypogonadism (i.e., low testosterone). This article is organized into several sections addressing specific questions related to the topic: (1) How is hypogonadism defined? (2) What is normal testosterone levels in men? (3) Why is testosterone so critical to athletes-exercisers? (3) What are situations inducing exercise hypogonadism? (4) Dysfunction or adaption-regulatory adjustment? (5) What are actions to deal with low testosterone in athletes-exercisers? and (6) Summary, conclusions and perspective.

## How is Hypogonadism Defined?

Hypogonadism is the medical term for decreased functional activity of the gonads. Male hypogonadism is characterized by a deficiency in the production of the critical male reproductive hormone testosterone from the testicles (8–10).

**Figure 1:**
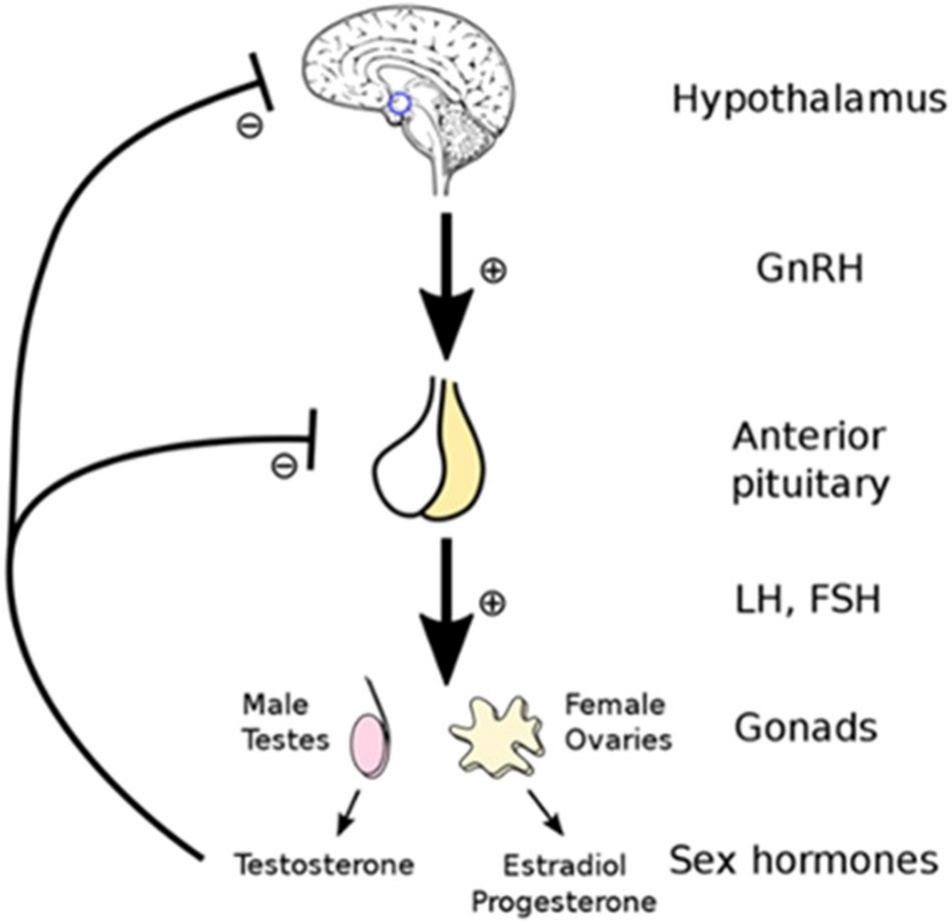
Testosterone production is controlled by the hypothalamic -pituitary-gonadal (HPG) regulatory axis which involves the hormones gonadotrophin-releasing hormone (GnRH), luteinizing hormones (LH), and follicle-stimulating hormones (FSH). Reprinted with permission: Artoria2e5 [CC BY 3.0 (https://creativecommons.org/licenses/by/3.0)].

Testosterone production is regulated by the hypothalamic-pituitary-gonadal (HPG) axis which involves the hypothalamic hormone gonadotrophin-releasing hormone (GnRH), and the pituitary hormones luteinizing hormones (LH), and follicle-stimulating hormones (FSH) (see Figure 1). As such, the low testosterone levels of hypogonadism may be due to testicular production or abnormalities in the HPG regulatory axis (11).

Specifically, two basic clinical types of male hypogonadism exist (9):

- *Primary*—This type of hypogonadism—also known as primary testicular failure—originates from a problem in the testicles. This can lead to what is termed hypergonadotropic hypogonadism, an impaired response of the gonads to GnRH, or LH and FSH stimuli (10).
- *Secondary*—This type of hypogonadism indicates a problem in the hypothalamus or the pituitary gland—which signals the testicles to produce testosterone. That is, within the HPG regulatory axis GnRH or LH and FSH are not produced adequately. In secondary hypogonadism, the testicles are generally normal in function. Another term used for this hypogonadism form is hypogonadotropic hypogonadism (10).

Either type of hypogonadism may be caused by an inherited (congenital) trait or something that occurs during a persons' lifespan (acquired). Relative to the discussion of this article, exercise hypogonadism would be viewed as acquired. Table 1 presents some of the major health-related clinical conditions associated with primary and secondary hypogonadism development (9, 12, 13).

**Table 1:** The major clinical conditions associated with the development of primary and secondary hypogonadism in men (9, 12).

**Primary hypogonadism conditions**
Klinefelter's syndrome Undescended testicles Mumps orchitis Hemochromatosis Injury to the testicles Cancer treatment Normal aging (*andropause*)
**Secondary hypogonadism conditions**
Kallmann syndrome Pituitary disorders Inflammatory disease HIV/AIDS Medications/pharmaceuticals Obesity Stress-induced hypogonadism

## What is Normal Testosterone Levels in Men?

The clinical reference range for normal testosterone levels in healthy, non-obese human males varies slightly based upon which scientific source is examined, and is relative to the age of the males. For example, Table 2 presents the reference values reported by the Mayo Clinic (14), as well as from the innovative study by Travison et al. which attempted to develop harmonized reference values of testosterone for wide clinical use (15). The values presented from these two sources are similar and overlapping but are not exactly the same.

**Table 2:** The reference range for clinical assessment of testosterone from select sources for non-obese men (i.e., Body Mass Index [BMI] <30 kg•m^2^).

**Source**	Total testosterone^**^	**Free testosterone**
Mayo Clinical Laboratories (14)	17–18 years: 300–1,200 ng/dl ≥ 19 years: 240-950 ng/dl	20<25 years: 5.25–20.7 ng/dl 25<30 years: 5.05–19.8 ng/dl 30<35 years: 4.85–19.0 ng/d 35<40 years: 4.65–18.1 ng/dl 40<45 years: 4.46–17.1 ng/dl 45<50 years: 4.26–16.4 ng/dl 50<55 years: 4.06–15.6 ng/dl 55<60 years: 3.87–14.7 ng/dl 60<65 years: 3.67–13.9 ng/dl
Travison et al. (15)	19–39 years: 304–850 ng/dl^*^ 40–49 years: 273–839 ng/dl 50–59 years: 256–839 ng/dl 60–69 years: 254–839 ng/dl	

* *(5th−95th percentile)*.

** *Total testosterone encompasses the free and carrier-protein bound levels of the hormone, while free refers only to that portion not bound to a carrier-protein in the circulation*.

**Figure 2:**
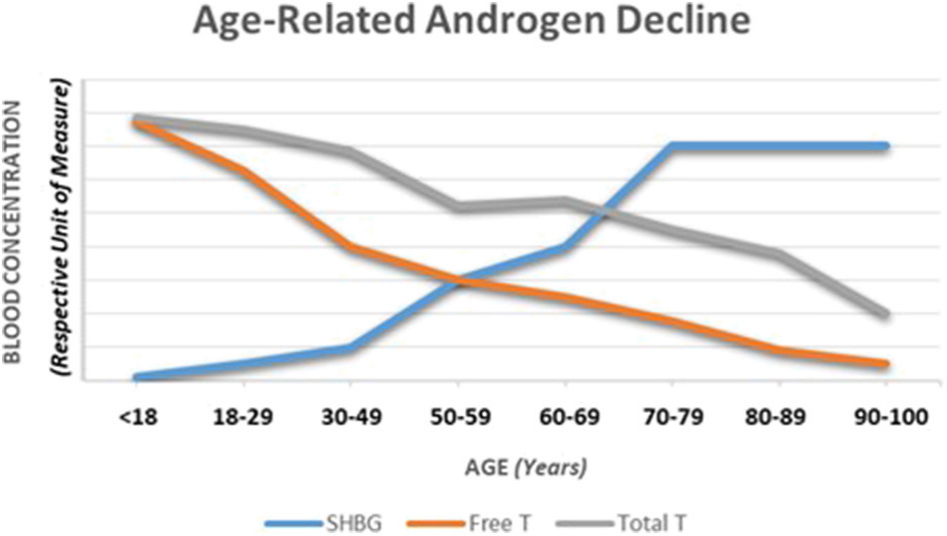
A depiction of the typical changes observed in total and free testosterone as well as sex hormone-binding globulin over the course of a male lifespan. Adapted from information provided in references 16, 28, and 49.

As noted in Table 2, testosterone can be expressed as either in total or free forms. The free, unbound form represents typically 1.5–2.0% (males) of the total hormonal amount circulating in the blood. The remainder is bound to carrier proteins; about 65% to sex hormone-binding globulin (SHBG) and 33% bound weakly to albumin (9, 10, 12, 16). The free and albumin-bound forms of testosterone constitute what is referred to as bioavailable testosterone (i.e., able to interact with androgenic receptors at target tissues). As males age, the amount of total and free forms of testosterone in the circulation change as does SHBG (see Figure 2) leading to a gradual overall reduction in the hormone forms in the blood; see subsequent section for discussion on the phenomena of andropause in males.

### What About Exercising Men?

Perhaps more pertinent to exercising or athletic males are the recent findings reported by Handelsman et al. in *Endocrine Reviews* (16). These authors did an exhaustive examination of the available research literature as well as the extensive database from the International Association of Athletics Federation (IAAF) on athletes who have competed over many years at elite levels in track and field (i.e., athletics). They concluded that a reference range (95%) of 223–849 ng/dl (7.7–29.4 nmol/L) existed in healthy adult athletic men, and 0–144 ng/dl (0–5.0 nmol/L) in athletic women. To this last point, the Handelsman et al. reference range, however, is an issue of some contention as it has been challenged by the legal team involved with the Caster Semenya vs. IAAF case at the Tribunal Arbitral du Sport (Court of Arbitration for Sport) concerning male and female categorical standards for acceptable gender-based testosterone levels (16, 17).

Nevertheless, and importantly though, the universal agreement does not currently exist in the world-wide medical community on what is precisely normal testosterone levels. Furthermore, the clinical definition of what constituents “low testosterone” and the diagnostic threshold for hypogonadism diagnosis varies too. To this last point, Table 3 illustrates this lack of agreement as it displays what might constitute hypogonadism based upon testosterone levels as defined by several medical organizations (18).

**Table 3:** Testosterone threshold levels for diagnosis of hypogonadism and, or androgen deficiency (also called testosterone deficiency) (18).

**Organization**	**Total testosterone**	**Free testosterone**
European academy of andrology International society of andrology International society for the study of the aging male (2009)	<350 ng/dl (12.1 nmol/l)	<65 pg/ml (<225 pmol/l)
The endocrine society (2010)	<300 ng/dl (<10.4 nmol/l)	<50–90 pg/ml (173–312 pmol/l)
European association of urology (2012)	<350 ng/dl (12.1 nmol/l)	<84 pg/ml (<243 pmol/l)
Expert opinion (2014)	<400 ng/dl (13.9 nmol/l)	80–100 pg/ml (277–347 pmol/l)

It should be noted, for some clinicians and medical professional groups, hypogonadism is characterized by not just low testosterone but includes at least one clinical sign or symptom (9). Overt signs of hypogonadism include absence or regression of secondary sex characteristics, anemia, muscle wasting, reduced bone mass or bone mineral density, oligospermia, and abdominal adiposity. Symptoms include sexual dysfunction (e.g., erectile dysfunction, reduced libido, diminished penile sensation, difficulty attaining orgasm, and reduced ejaculate), reduced energy, and stamina, depressed mood, increased irritability, difficulty concentrating, changes in cholesterol levels, anemia, osteoporosis, and hot flushes (9, 12, 13).

In the absence of any of the clinical signs or symptoms, the presence of low testosterone alone may lead to a diagnosis of “androgen deficiency” (also called testosterone deficiency) and not definitively hypogonadism. That said, nonetheless, many leading medical resources define hypogonadism based solely on the presence of low circulating testosterone (9, 12).

### How is Exercise Hypogonadism Defined?

The term exercise hypogonadism has been applied in a number of exercise studies in which low testosterone levels are reported, but in doing so investigators have seldom applied the criteria as outlined in Table 3 for their defining of hypogonadism. In fact, other criteria have been used, for example:

- If the study was cross-sectional in design there has typically been a matched-control group (sedentary) to whom the exercising males are compared to determine if testosterone status is low or reduced;
- If the study research design was prospective, or longitudinal in approach the exercising males are usually compared to themselves at some point in time before training when their testosterone was not affected; and,
- In some studies, the testosterone levels of exercising males have been compared to a clinical reference range set of values to determine testosterone status.

Additionally, some research groups have been hesitant to use the term hypogonadism altogether, and have referred to the exercising males as having states of “low testosterone,” “testosterone deficiency,” or “androgen deficiency” (6, 19–22). Although, again what constitutes a *low* or *deficiency* level has not been clearly defined or have used endocrine standards per professional organization guidelines as noted in see Table 3. And, while not using the term hypogonadism strictly some published exercise reports have alluded to consequences associated with hypogonadal states from there testosterone findings.

In short, there is a lack of consistency in the exercise literature determining what exactly constitutes exercise hypogonadism. Additionally, few investigators have attempted to set or use a threshold, or cut-point value to denote when testosterone levels are reduced enough to use the “exercise hypogonadism” distinction. Regardless of the terms used to refer to testosterone levels in exercising men, it is important to note that even were testosterone is reduced, for many of these individuals it is low but within the normal range and seldom found to reach clinical definitions of hypogonadism (Table 3). Although, reports of sub-clinical findings and testosterone levels well below those established for clinical hypogonadism exist (23–25).

Notably, in 2005 Hackney and associates did outline criteria for the level of testosterone reduction necessary to denote an athlete having what they termed the “Exercise Hypogonadal Male Condition” (see later discussion) (19, 26). These investigators suggested persistent reductions of 25–50% or greater in testosterone were necessary for this distinction as a relative form of hypogonadism.

## Why is Testosterone so Critical to Athletes-Exercisers?

Throughout the male lifespan, testosterone plays a critical role in sexual, cognitive, and body morphology development. The most visible effects of rising testosterone levels begin in the pre-pubertal stage for males. During this time a multitude of physiological changes occur; e.g., body odor develops, oiliness of the skin and hair increase, acne develops, accelerated growth spurts occur, and pubic, early facial, and axillary hair grow. The pubertal effects also include enlargement of the sebaceous glands, penis enlargement, increased libido, increased frequency of erections, increased muscle mass development, deepening of the voice, increased height, bone maturation, loss of scalp hair, and growth of facial, chest, leg, and axillary hair. Several, but not all of these essential effects and influences continue into adulthood (27, 28).

Many aspects of the above influences affect the male physiology advantageous for sporting performance. Perhaps the most striking being the anabolic action of testosterone on protein turnover and the potential to develop muscle accretion (16, 29, 30). Although, the process is not solely dependent upon anabolic hormones such as testosterone (31). With proper exercise training regimens, such muscular development can lead to enhanced strength and power. Additionally, testosterone exhibits positive effects on erythropoiesis and hemoglobin concentrations (16). The latter in turn can facilitate the oxygen content capacity of the blood and maximal aerobic capacity (VO_2max_) (16, 32). All of these components, strength-power-oxygen content-VO_2max_, are critical factors in the performance of a multitude of sporting activities and essential elements in the exercise training adaptation process (16, 32, 33).

Unlike women who experience a rapid decline in sex hormone levels during menopause, men experience a slow, continuous decline in testosterone levels over time (see Figure 2). The term “andropause” is sometimes used to denote this hormonal change. As testosterone levels slowly reflect this decline with aging, a form of hypogonadism can develop and is sometimes referred to as the partial androgen deficiency of the aging male (PADAM) (34). In older athletic men who display reduced levels of testosterone, this aging event could be a partial contributor to hormonal change. But, research examining older men who are exercisers with low testosterone compared to sedentary controls still show reductions in their testosterone levels compared to age-matched controls, although the amount of research on this topic is extremely limited (35).

When male athletes develop low testosterone-hypogonadism the physiological and psychological consequences and side effects are variable. Some studies report serious negative consequences and other studies reporting no negative effects whatsoever (21, 23, 25, 36–38). This lack of consistency in studies may relate to the degree of reduction in testosterone observed and, or the scope of health-related outcomes monitored within these studies (39). Examples of the negative psychophysiological consequences typically reported are given in Table 4 (39).

**Table 4:** Signs and symptoms of low testosterone and hypogonadism typically reported by men, non-athletes as well as athletes (39).

**Low testosterone—hypogonadism consequences**
Decreasing physical performance Sleep disturbances Lethargy Decreased motivation Decreased libido Sexual dysfunction Spermatogenesis abnormalities Muscle mass loss Sperm abnormalities Bone mineral density loss Depression

## What are Situations Inducing Exercise Hypogonadism?

### Background

The systematic and scientific study of the influence of exercise on testosterone levels in human males began in the 1970's. Animal-based research had pre-dated this period considerably, and human anabolic steroid “doping experiments” by athletes-coaches also occurred before this period. Although the evidence of the latter actually occurring was withheld from public and scientific scrutiny due to legality and ethical violation issues for many decades. Perhaps the first systematic exercise study on humans was performed by the late Dr. John Sutton of Australia in the 1970's. He and his associates published an article on the testosterone response in men and women to acute submaximal and maximal exercise sessions (40). They reported that maximal exercise increased testosterone levels, and with this finding, a cornucopia of studies was begun by the scientific community examining testosterone, exercise, and training adaptations.

By the mid-to-late 1980's, several key studies were published which reported men involved with endurance exercise training had substantially lower resting testosterone levels (41–44), and or HPG axis disruptions [potentially affecting testosterone levels (historically the vast majority of these studies have examined total testosterone; although, a few research groups have addressed free testosterone too and found both total and free to be reduced)] (45). These studies involved distance runners, and at the time these investigators did not speculate on the causation of the low resting testosterone. Nonetheless, these studies served as the basis for subsequent work which did attempt to examine causality (see following discussions).

In the context of exercise endocrinology, it is important to understand the distinction between the effects of an acute exercise session on hormones, and the more chronic effect of exercise training on hormones. In the acute scenario, nearly all forms of exercise provoke changes in circulating hormone concentrations—almost universally being increased levels, which tend to be proportional to the intensity at which the exercise is conducted and, or the extent of the exercise duration. Although the mode of exercise utilized creates some variance in the degree of response (e.g., swimming vs. running, vs. weight lifting) (46–48). Additionally, some hormones do display a “threshold” level of exercise volume (i.e., intensity X duration of exercise sessions) be achieved before a response is detected in the blood (49). These acute exercise-induced changes abate relatively quickly during the recovery period unless the exercise session is extremely excessive (e.g., hours) in duration (49). Table 5 provides a basic summary of the generalized effects of exercise on the major hormones associated with research-clinical interests in the area of sports physiology and exercise.

**Table 5:** The generalized hormonal responses to exercise (e.g., resting-basal levels compared to after an exercise session [~immediately] of the respective exercise type).

**Hormone**	**Physiological actions**	**Exercise type—response**		
		**High intensity (e.g., HIIT)**	**Endurance exercise (>60 min)**	**Resistance exercise**
ACTH	Adrendo-regulatory	↑	↑	↑
ADH	Hydration, fluid balance	↑	↑	↑, ↓, ↔
Aldosterone	Hydration, fluid balance	↑	↑	↑
Catecholamines (adrenaline, noradrenaline)	Catabolic (e.g., lipolysis, glycogenolysis), cardio-regulatory	↑	↑	↑
Cortisol	Catabolic (e.g., lipolysis, gluconeogenesis), stress reactivity	↑ >60%VO_2max_	↑ >60%VO_2max_	↑
DHEA	Anabolic	↑	↑	↑
Estradiol-β-17	Bone metabolism, catabolic (e.g., lipolysis), reproductive function	↑	↑	↑
			↓ if excessive	
FSH—LH	Reproductive function	↑, ↓, ↔	↑, ↓, ↔	↑, ↓, ↔
Glucagon	Glucoregulatory	↑	↑	↑
Growth Hormone	Anabolic (e.g., myoplasticity), Catabolic (e.g., lipolysis)	↑	↑	↑
Insulin	Glucoregulatory, anabolic	↓	↓	↑, ↓, ↔
IGF-1	Anabolic	↑, ↔	↑, ↔	↑, ↔
Leptin	Satiety, reproductive function	↑, ↓, ↔	↑, ↓, ↔	↑, ↓, ↔
Parathyroid	Calcium metabolism	↑	↑	↔
Prolactin	Immune function, stress reactivity	↑	↑	↑
Progesterone	Reproductive function	↑	↑	↑
Testosterone	Anabolic (e.g., myoplasticity), reproductive function	↑	↑ ↓ if excessive	↑
T_4_–T_3_	Calorigenesis, endo-permissive actions	↑, ↓, ↔	↑, ↓, ↔	↑, ↓, ↔
TSH	Thyroid-regulatory	↑, ↓, ↔	↑, ↓, ↔	↑, ↓, ↔
Vitamin D	Calcium metabolism	↔, ?	↑	↔, ?

*HIIT, high intensity interval training exercise; ACTH, adrenocorticotropic hormone; ADH, antidiuretic hormone (vasopressin); DHEA, dehydroepiandrosterone; FSH, follicle-stimulating hormone; LH, luteinizing hormone; T_4_, thyroxine; T_3_, triiodothyronine; TSH, thyroid-stimulating hormone; VO_2max_, maximal oxygen uptake; ↑ = increase; ↓ = decrease; ↔ = no change; ? = unknown*.

Conversely, when examining the chronic effect of exercise one can examine resting (basal) effects and, or responses to a subsequent exercise session after some period of training has been performed. Resting, basal hormone levels after substantial exercise training are commonly unchanged, increased slightly or perhaps reduced slightly. Relative to the latter, the “basement effect” phenomena prevent some aspects of detectable reductions being observed; that is a hormone value near zero cannot be reduced substantial further (50). In response to performing an acute exercise session following chronic exercise training, many hormone responses are reduced when compared to performing a similar exercise session before the training intervention; although the direction (↑ or ↓) of the hormonal change remains the same. These reduced responses tend to be a function of reduced stress reactivity to any given exercise bout and due to improved target tissue sensitivity as a training adaptation (51, 52). In general, these acute-chronic exercise endocrine principles for hormonal response hold true for the reproductive and non-reproductive hormones (52). Finally, and importantly to the present discussion, in most clinical diagnosis settings, much of the assessment and detection of reproductive dysfunction relies on evaluating hormonal status in a resting, basal condition and not in response to an exercise session (53). In such assessments, the gold standard, biological fluid for measurement is blood serum or plasma. Other fluids are occasionally assessed such as saliva or sweat; but, these fluids can produce a variance in outcomes. For example, Adebero and associates compared salivary and serum concentrations of testosterone and cortisol at rest and in response to intense exercise in boys and men; and, found testosterone was reduced post-exercise in serum but not in saliva (54). VanBruggen and colleagues have attributed such discrepancy in blood-saliva findings as being due to changes in hormonal diffusion rates into the salivary gland-saliva being effected by the physiological consequences of exercise (e.g., plasma volumes shifts, changing hormonal concentration gradients) (55).

### Overtraining Syndrome

**Figure 3:**
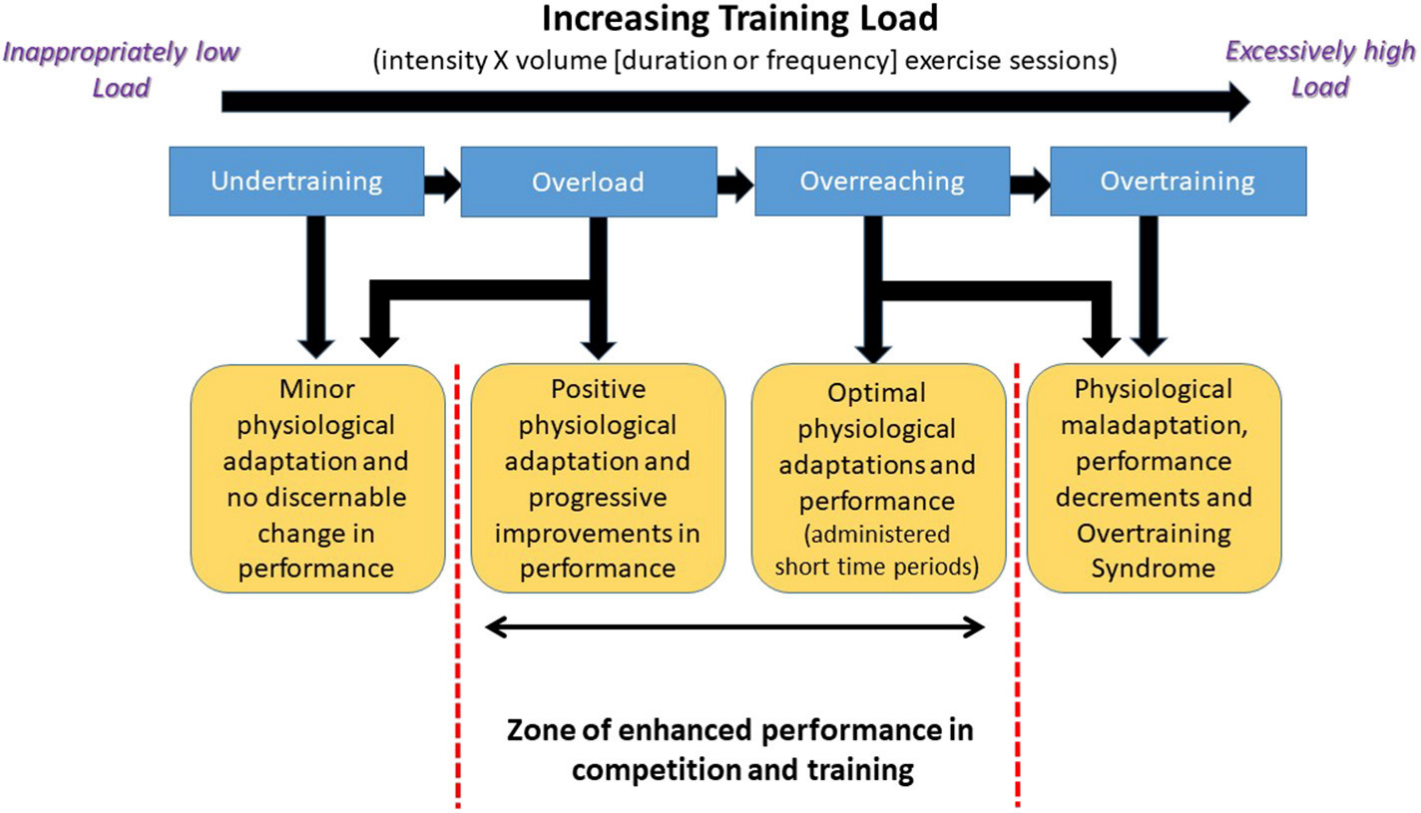
Schematic representation of the progression in exercise training load that leads to the development of the Overtraining Syndrome in athletes. Adapted from information provided in reference (61). Used with permission.

In their extensive review, Kuiper and Keizer, provide a thorough historical background on the use of the term overtraining, and commentary on the early research in the topic. Many coaches and exercise scientists would be surprised to find that this topic has been recognized and discussed for nearly 100 years (56). That said, there have been attempts to change the language and nomenclature used in describing the issue and shift the explanations to some degree in the operational definitions of the terms associated with it over the decades (57). For example, in their recent innovative EROS study (Endocrine and Metabolic Responses on Overtraining Syndrome), Cadegiani and Kater proposed a new designation of “Paradoxical Deconditioning Syndrome” rather than Overtraining Syndrome (58, 59). Nevertheless, regardless of what is called, for the most part, the indicators of the condition are essentially the same topical area as when first mentioned in a 1939 sports medicine article by Jezler (60). To aid the reader, with what constituents the progression from normal and appropriate levels of training to overtraining Figure 3 (61) is provided and references 56 and 61 are recommended reading.

Because of testosterone's critical physiological role, early in the pursue of exercise adaptation research investigators began proposing the question—“Can monitoring of circulating testosterone changes serve as a viable biomarker of training adaptation?”. Research work in the late 1970's and early 1980's by groups of various Scandinavian and Baltic researchers reported intensive exercise sessions and training loads resulted in substantial reductions in blood testosterone (62–67). These numerous findings led to Aldercreutz and associates in 1986 releasing their seminal paper suggesting that testosterone, cortisol and, or the ratio of the two (T:C ratio) could be used as a means of accessing “overstrain” (i.e., overtraining) in an athlete and monitoring whether their training was progressing advantageously (68). Shortly thereafter, reports began appearing of overtrained athletes having low testosterone, and in some cases elevated cortisol which was associated with the testosterone reductions (69–73).

To that end, over the next 30 years, a great number of studies reported with increasingly heavy training loads testosterone becomes reduced and this typically coincides with performance stagnation or declines in athletes as they become overtrained (i.e., primarily males; see review articles—references (74–76)); although, this is not a universal finding (25). Table 6 displays some of the signs, symptoms and health consequences of athletes diagnosed as having the Overtraining Syndrome. The syndrome results in a chronic under-performance, negative health consequences (see Table 6), and typically can end or curtail an athlete's competitive season (56, 57, 77). The development of the Overtraining Syndrome has been reported in a multitude of sports, regardless of the emphasis on training modality employed (e.g., runners vs. weight lifters vs. tennis players) although the specific symptoms and frequency of select symptoms can be somewhat sports specific (74, 75).

**Table 6:** Symptoms and characteristics displayed by athletes (male) who are overtrained (74, 75).

**Parasympathetic alterations^*a*^**	**Sympathetic alterations^*b*^**	**Other-combined^*c*^**
Fatigue	Insomnia	Declining performance
Depression	Irritability	Anorexia—weight loss
Bradycardia	Agitation	Lack of mental concentration
Loss of motivation	Tachycardia	Heavy, sore stiff muscles
Hypotension	Hypertension	Anxiety
Abnormal heart rate during recovery	Restlessness	Awaking unrefreshed
	Increased basal metabolic rate	Endocrine abnormalities (e.g., low testosterone, elevated cortisol, low thyroid hormones)

a *Typically symptoms more associated with more endurance-based sports*.

b *Typically symptoms more associated with strength-power based sports*.

c *Symptoms common to either form of sports activities*.

Researchers have proposed two major rationales and mechanisms for testosterone reductions observed with overtraining; (1) testosterone production being disrupted by inhibitory factors such as other hormones in a stress response cascade; and, (2) inadequate energy intake disruption of the HPG axis regulatory function.

Relative to the first mechanism, Doerr and Pirke, as well as Cummings and associates, demonstrated blood cortisol elevations disrupt testosterone production peripherally at the gonad (testes) when cortisol levels were elevated (78, 79). There are numerous research studies reporting findings of exercise-induced short-term increases in cortisol levels (see review articles—references (74, 78)), as well as these acute elevations in cortisol from an exercise session being associated with decreases in testosterone (72, 80, 81). Furthermore, evidence exists for circulating testosterone and cortisol to be negatively associated with athletes even in the resting, basal state (82). In these scenarios the inhibitory effect of cortisol appears twofold; i.e., to impact LH and FSH via GnRH suppression as well as a compromise of Leydig cell function via direct steroidogenesis inhibition (79, 83). Prolactin is another hormone that can induce reductions in testosterone levels, and this hormone's release is also stimulated by exercise (see review article—(84)). The evidence convincingly shows elevated prolactin concentrations inhibit the secretion of GnRH, thereby decreasing the secretion of gonadotropins (LH, FSH) and affecting the central aspects of the HPG axis (85). Additionally, prolactin may also inhibit the action of gonadotropins on the gonads directly (86). Acute exercise-induced elevations in prolactin have been associated with testosterone reductions (87), as have training-induced increases in resting, basal prolactin associated with testosterone reductions (73, 88); but the latter is not universally reported (41, 89).

Nevertheless, resting hypercortisolemic or hyperprolactinemic states are not frequently found in athletes, but consistent daily exercise sessions could create frequent transient periods of such hyper-exposure during an actual exercise session as well as for extended periods in the recovery from such exercise sessions (80, 84, 90, 91).

In the case of the second proposed mechanism, several researchers' decades ago demonstrated short- and long-term caloric deficient results in testosterone reductions in men (92–94). It is well-recognized that a common finding is overtrained athletics is weight loss and suppressed appetite/anorexic tendencies (56, 61). The effect of inadequate caloric intake on testosterone seems more related to central HPG axis suppression than direct action at the testes as both LH and FSH levels become reduced in such scenarios. For example, Bergendahl et al. (95) found such gonadotrophin reductions were driven by suppressed GnRH release by the hypothalamus. Recently, Wong and associates (96) propose this dysfunction likely involves hypothalamic suppression due to dysregulation of leptin, ghrelin, and pro-inflammatory cytokines. The gonadal axis suppression transient and the axis functional, as the effect, can be reversible with weight gain; although the rate of testosterone returning to normal seems highly individualistic (96–98).

### Weight Restricted Sports

Historically, one of the exercise activities, where dramatic testosterone reductions were first reported in athletes, involved the sport of wrestling (i.e., Olympic free-style, Greco-Roman, and or American scholastic-collegiate forms). For example, nearly 40 years ago researchers described substantial testosterone reductions in adult male wrestlers during their competitive season compared to their off-season period (99). Subsequent reports by numerous other investigators substantiated these findings not only in wrestlers but other weight-restricted sports too (100–104).

Mechanistically the reason for this reduction in testosterone most likely is related to the practice of many athletes in these sports to use extreme weight loss tactics (e.g., semi-starvation) in attempting to reach a specific competitive weigh category. That is, their reduced caloric intakes plus high exercise expenditures lead to extreme negative energy balances and an HPG axis suppression—specifically, a hypogonadotropic hypogonadism state development—see preceding section discussion (105). Although this occurrence also seems highly reversible as a resumption of appropriate caloric intake reverts the HPG axis function relatively quickly (96, 98, 105).

### Contact—Combative Sports

It is well-known traumatic brain injuries (TBI), such as concussions, can result in the development of low testosterone; specifically, a secondary hypogonadism usually develops due to a pituitary dysfunction (106, 107). A great deal of contemporary research has focused on American football and these type injuries as investigations on professional and collegiate athletes who have experienced multiple concussions show serious long term negative health consequences of such repeated head traumas (108). But, there are a number of sporting activities which results in participants being at an increased risk for the development of some form of TBI. Sporting activities categorized as “contact sports” (some of which are also referred to as combative sports) present the greatest risk—boxing, kickboxing, karate, taekwondo, aikido, jujitsu, judo, rugby, and Australian football. While sporting activities such as these have a greater risk for TBI exposure, a multitude of sports even if not specifically categorized as a contact-combative can result in an athlete developing a TBI (e.g., wrestling discussed in prior section or football [soccer]). It is important for clinicians to examine an athlete's medical history for TBI events if they detect the presence of low testosterone.

### Male Triad/RED-S

The Female Athlete Triad refers to a medical condition that is a constellation of three clinical entities: menstrual dysfunction, low energy availability (with or without an eating disorder), and decreased bone mineral density (7). The Triad term for this disorder was first coined by the American College of Sports Medicine in 1992 after many experts in the field had noticed a pattern among adolescent and young adult female athletes. Evidence from landmark work by Dr. Anne Loucks demonstrated that the etiological cause of the Triad in women was a persistent state of low energy availability (109).

Relative to this discussion, it is important to define the term “energy availability”. Energy availability refers to the amount of energy leftover and available for your body's functions after the energy expended for daily exercise training is subtracted from the energy taken in from daily caloric intake from food. In other words, in its most basic form:

$$\begin{array}{l} \text{Energy Availability}=\text{Dietary Energy Intake }\left(\text{food}\right)\\ \;-\text{ Exercise Energy Expenditure} \end{array}$$

Extensive research in females has identified low energy availability cut points indicative of risk level for the development of physiological and performance disturbances associated with the Triad. These cut-points are: at risk = ≤30 kcal/kg lean body mass (LBM); moderate risk = 30–45 kcal/kg LBM; and no risk = ≥45 kcal/kg LBM (109). Whether male athletes share the same risk factor cut points is currently unknown, and is an issue of debate (109).

Recently, DeSouza and associates have proposed an expansion of the scope of the Triad condition and use of the term to encompass not only the historic population of women but also males (110). Interestingly, earlier researchers had drawn an analogy between the development of menstrual disruptions in exercising women and the observation of low testosterone in men but had never applied the Triad terminology to men (111, 112).

While the state of low energy availability (LEA) produces a myriad of physiological consequences in women and supposedly men, it is associated specifically with the development of low testosterone in men (110). The mechanism for such a change appears consistent with earlier work supporting the development of hypogonadotropic hypogonadism as with extensive caloric deficient, weight loss and restricted food intake (see prior discussions). Historically the idea of caloric intake and energy status as being associated with the low testosterone in exercising men was alluded to in the 1980's but a systematic examination of the concept was not thoroughly pursued until recent times (44, 101).

It is now recognized that a state of LEA not only can lead to the Triad condition but also the “Reduced Energy Deficiency in Sports” [RED-S] condition. RED-S was designated as a separate entity from the Triad by an International Olympic Committee medical commission group of clinicians; and, is found in men as well as women. RED-S is different from the Triad as it is viewed as more broad in scope. It is defined as impaired physiological function including but not limited to, metabolic rate, menstrual function, bone health, immunity, protein synthesis, and cardiovascular health caused by relative energy deficiency brought on by a state of LEA (113).

The common etiology and a certain degree of overlapping symptomology of the Triad/RED-S have caused some to question whether they truly represent two distinct conditions (114). That difference of opinion requires more research to be fully resolved. What is clear is a state of LEA can lead to low testosterone levels in men. Hooper and associates show this clearly in their cross-section studies where LEA was linked to low testosterone in distance runners and triathletes (115, 116). For a full discussion of the endocrinological impact of RED-S the reader is direct to the recent review article by Elliot-Sale and associates (117).

### Exercise Hypogonadal Male Condition

In 2005 Hackney and associates proposed the use of the term “Exercise Hypogonadal Male Condition” (EHMC) for exercise-trained men who showed lowered testosterone (19, 26). They based this recommendation upon work by their own and other research groups from the 1980's and 90's. This recommended terminology was targeted to exercising men who displayed functional hypogonadotropic hypogonadism and met certain criteria and was not intended for universal application to all exercising men with low testosterone. The key characteristics and traits of EHMC laid out by this research group were (19, 26):

- These men had testosterone levels at least 25% to 50% lower than expected for their age.
- The lowered testosterone levels did not appear to be a transient phenomenon related to the acute stress-strain of exercise training.
- The men were not experiencing a performance decrement or lack of motivation (i.e., overtrained).
- They had not experienced a major bodyweight loss in recent months.
- The men had a history of early involvement in sports resulting in them have many years of nearly daily exercise activity.
- The modality of exercise and training most frequency associated involved high volume endurance activities such as running, triathlons, cycling cross-country skiing, and race walking.

Regrettably, there has been some confusion in the research community concerning the EHMC terminology. That is, many researchers have assumed that the EHMC connotation was the same as exercising men displaying overtraining or Triad/RED-S (… *etcetera*) related to the lowered testosterone. EHMC as originally proposed over 15 years ago was for a different condition and one representing a potential adaptive response in the reproductive system HPG axis from chronic, long-term exercise exposure (see the following section). This point seems to have been overlooked and as such use of the EHMC term has been applied incorrectly, or entirely ignored altogether as a categorical distinction for exercising men with persistent low, resting testosterone.

### Special Considerations

Regrettably, it is nearly impossible to address the topic of testosterone and sporting activities without mentioning anabolic-androgenic steroids (AAS) and doping by athletes. AAS, which are the synthetically produced variants of naturally occurring testosterone, have been associated with certain sports for decades. While these products have valid and legitimate medical uses they are banned or prohibited by sports governing bodies for creating an unfair physiologic advantage (16, 21, 52). There are a great number of side-effects of AAS use, and the complications are variable and individually specific; but, one common outcome is a variant of hypogonadism developing (118). The hypogonadism in this situation can be during active AAS use as well as a long-term side effect once usage has ceased (118). It is advisable when considering some of the potential causes of hypogonadism in athletes, as discussed in prior sections that researchers and clinicians rule out AAS use as likely causative factor.

## Dysfunction or Adaptation- Regulatory Adjustment?

Much of the current contemporary research focuses on the role of energy balance and energy availability on the development of exercise relative hypogonadism. Ample evidence points to a negative energy balance, caloric restriction or a state of LEA leading to low testosterone development. This form of exercise hypogonadism-low testosterone is a transient phenomenon that can be abated with appropriate interventions (see the following section). As noted though, it has been proposed that not all formed of exercise hypogonadism-low testosterone fall into this category (119). Specifically for some men, this occurrence may represent an adaptation within the reproductive system due to their persistent and chronic exposure to large volumes of exercise training regularly; which has been termed the EHMC state.

Evidence supports that the reduction in testosterone inducing a form of exercise relative hypogonadism is detrimental in the case of men experiencing the overtraining and, or Triad/RED-S. These individuals have compromised health and physical performance that results in an inability to compete at their maximal potential, optimal level. These individuals are experiencing a classic endocrine dysfunction.

Conversely, men denoted as experiencing EHMC does not show the same compromised health and performance issues; and report no overt adverse signs or symptoms of poor health (although, granted not all studies examining EHMC men have thoroughly examined all aspects of their subject's health profile). These individuals do not appear to be experiencing an endocrine dysfunction, but it is hypothesized their condition reflects an adaptation-regulatory adjustment in the HPG axis in which a new set-point for what is a “normal” level of testosterone develops due to their chronic, regular exercise training, a view speculated on as well by other research groups (120).

Such a premise is in line with anthropological research and the energy constraint model as outlined by Pontzer (121). This model of Pontzer posits that total energy expenditure (TEE) is maintained within a narrow range. As daily physical activity increases, other components of daily energy expenditure are reduced to keep TEE in check. Non-essential expenditure would be expected to decrease first; essential activity would be spared unless physical activity workload becomes too excessive. Subsequently, moving from a sedentary to a chronic active lifestyle leads to a persistent downregulation of non-essential expenditures including reduced inflammation, reduced hypothalamic-pituitary-adrenal axis, and sympathetic nervous system reactivity, as well as reduced reproductive hormone levels and HPG axis function. Collectively these reductions lower the risk for a broad range of chronic diseases (e.g., cardiovascular disease; T2D, Type 2 diabetes) (121). In support of this model and the effect on reproductive function, Raichlen associates (122) found the Hadza, a hunter-gatherer population in northern Tanzania, where men accumulate nearly 2 h of moderate and vigorous physical activity daily, have testosterone concentrations roughly 50% lower to those in comparable North American men. Likewise, Trumble et al. found the Tsimane men, Bolivian foragers-farmers with high levels of daily physical activity, display similar testosterone reduction (30–35% lower) (123). Furthermore, generally resting testosterone is also lower among men in physically active non-industrial populations compared with those in less active, industrialized countries (124). Collectively these studies did not report their populations to be in high-stress situations (e.g., famine, warfare) or having insufficient food-caloric availability; hence, these hormonal changes seemed adaptive consequences of their lifestyle (121). Similar long term reproductive hormonal adjustments could be occurring in men designated as experiencing EHMC.

**Figure 4:**
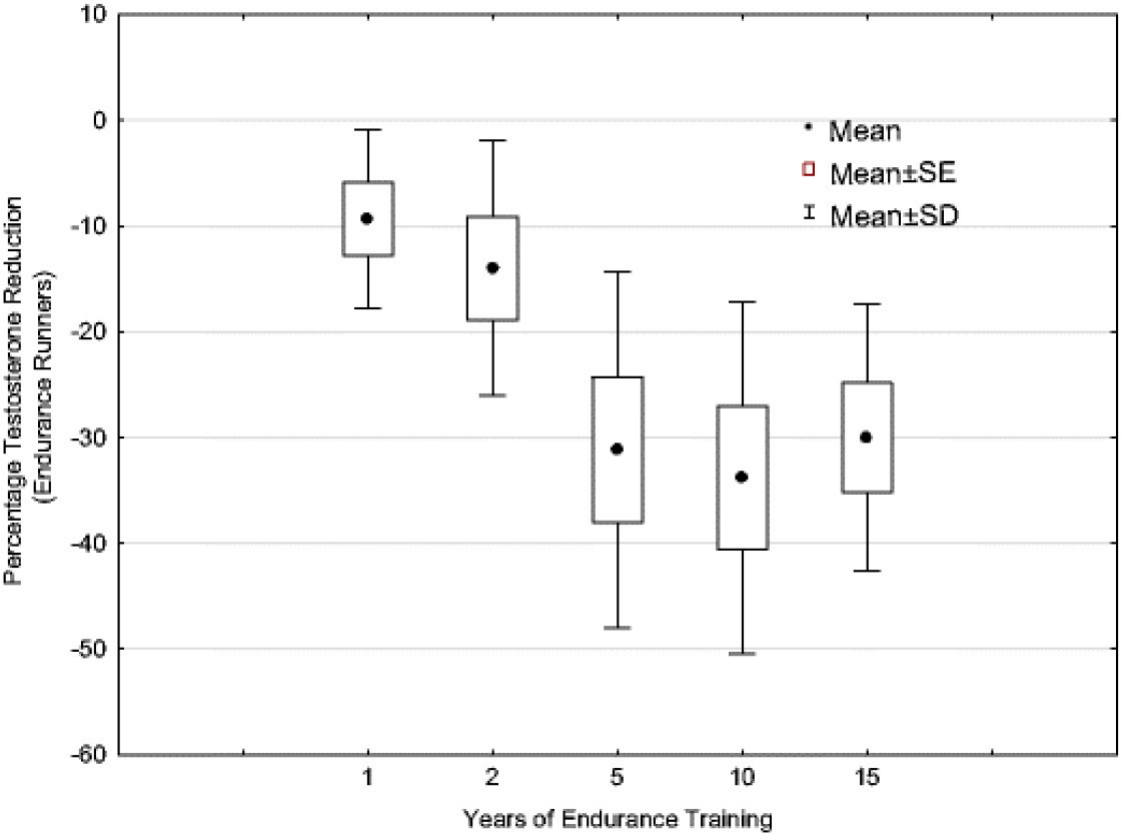
Testosterone levels of endurance-trained runners (age = 18–57 years) expressed as a percentage decrease of the non-exercising matched control subjects (*n* = 196). For years training: 1 year, *n* = 49; 2 years, *n* = 28; 5 years, *n* = 52; 10 years, *n* = 40; 15+ years, *n* = 27 (*N* = 196). Adapted from information provided in reference (35). Used with permission.

In support of this persistent downregulation phenomena as proposed by Pontzer, as a more chronic and regular physically active life-style develops, are the data presented in Figure 4 (24, 35). This figure illustrates that the longer an endurance athlete (i.e., runner) is engaged in consistent and chronic endurance training, the lower their resting testosterone becomes. These data are from a cross-sectional, longitudinal case-control study (*n* = 196) in which the result suggests the level of reductions plateaus at approximately 30–35%. In this study, and all runners met the criteria for EHMC as noted earlier. One could argue that these are perhaps LEA related occurrence, but it seems unlikely that chronic LEA over years would not precipitate a myriad of health problems associated with that condition and prevent these athletes from training, competing and being in a good physical condition/health (which was reported by all the participants). Furthermore, earlier work by our research group demonstrated that both pituitary and testicular responsiveness—sensitivity to drug challenges is attenuated in EHMC men and was substantially less than matched, sedentary control men (125, 126). This is inline and supported by the findings of Bobbert et al. who show hypothalamic-pituitary regulatory sensitivity is adjusted with exposure to endurance exercise training (127).

Granted this premise is postulated on limited evidence and research findings and as such the proposed etiology for EHMC development is a “working hypothesis;” but to that end, the entire scope of available research dealing directly with male exercise-related hypogonadism is extremely small in its totality and an evolving field of study. As stated by Sansone and associates, “*whether testosterone suppression is the result of a physiological adaptation to stress or an undesirable side effect of excessive training is a matter still open to debate”* and hence addition research on this important question needs to be pursued (128). That is, specifically researchers and clinicians need to address the questions within this statement and discern whether:

- The reduction in testosterone levels (and hypogonadism) are occurring as an undesirable side effect of exercise training, which suggests there are potentially harmful effects on the human physiology from performing chronic physical activity (*N.B.*, a line of thinking rarely discussed or mentioned in the exercise literature or media portrayal); or,
- If low testosterone (and hypogonadism) occurs as an adaptation response to the stress-stimulus of exercise training, would it be beneficial to leave such a condition untreated medically while athletes are training/competing? Or, would treatment of exercise-induced hypogonadism improve the relevant symptoms and overall health of the athlete (see Table 4)? (see the following section on treatment options).

These questions are open to discussion and future debate in the scientific and medical healthcare community.

## What are Actions to Deal With Low Testosterone in Athletes-Exercisers?

Normally, the medical standard of care for treatment of male hypogonadism typically centers on the use of pharmaceutical agents to address the existing low serum testosterone, either through exogenous testosterone administration or drugs to stimulate the production of testosterone via the HPG axis. However, athletes who are competing may not use such means according to the World Anti-Doping Agency (WADA; international agency regulating and monitoring doping in sports). Endogenous testosterone and gonadotropin stimulator agents (acting on the HPG axis) fall into the WADA “List of Prohibited Substances and Methods” (categories: S1 *Anabolic agents*; S2, *Peptide hormones, growth factors related substances, and mimetics*) which if used constitutes a doping violation by the athlete (129). WADA does have Therapeutic Use Exception (TUE) options which would allow for pharmacological intervention and treatment for health reasons, but the scenario by which hypogonadism-low testosterone occurs in men as a consequence of exercise training does not fit into the circumstances by which WADA would grant a TUE to an athlete (21). That is, in athletes hypogonadism-low testosterone develops due to the consequences of exercise training, and is not a preexisting medical condition, or considered an acquired disease outcome.

This leaves the athlete with more behavioral related options for treatment of their condition; i.e., if they choose to treat it. In the case of the overtraining-Triad/RED-S treatment seems warranted and advised, but in the case of weight-restricted sporting activities or EHMC scenarios, such actions may not always be chosen by the athlete. In 2018 Hooper and colleagues presented in *The Physician and Sportsmedicine* a thorough overview of treatment approaches. In short, they recommended treatment be centered on non-pharmacological strategies including nutritional intervention, and modifications in training volume to improve energy availability and support the normal hormonal function of the HPG axis in male athletes (21).

Even though testosterone or anabolic stimulator agents are not permitted by WADA, if the athlete is suffering from low body mineral density, bisphosphonates (also called diphosphonates; e.g., Fosamax®) can be a viable option as they are permitted as a treatment by WADA. Some research findings support an increase in total or free testosterone concentrations through legal supplements (for example; such as D-aspartic acid and fenugreek [*Trigonella foenum-graecum*]) (130, 131). But, the reported outcomes from such supplements are not substantial and as such is seldom recommended.

Copious internet sites advertise for male sexual performance enhancer supplements, which supposedly promote testosterone elevations (and increase libido). These sites are typically vague in what is the physiological mechanism for such actions, proprietary as to what are their “secret ingredients,” and heavy in testimonial accounts of efficacy; but lacking in scientific evidence. Furthermore, cases of such supplements containing substances that are banned by WADA have been reported; and ignorance of the contents of the supplement used by an athlete is not viewed as a viable excuse by WADA (132). Therefore, the athlete is not advised to experiment with supplements from such sites if they are actively competing and could be screened for doping violations.

Essentially, athletes and the clinicians working with them are left with few viable options for dealing with exercise-related hypogonadism and the consequences of the condition if they wish to stay within WADA guidelines. A review of the symptomology of hypogonadism, Table 4, clearly demonstrates that such individuals (athlete or non-athlete) would be compromised in many aspects of daily life and function.

Interestingly, much of the current, contemporary medical emphasis related to low testosterone and hypogonadism in exercising men has focused on bone health. This is a critically important concern, but the other consequences as noted can also substantially impact on overall health and quality of life in an individual, and as such should not be ignored by healthcare providers.

## Summary, Conclusions, and Perspective

The renewed interest and explosion of new research on exercising men and hypogonadism development seems long overdue; as the topic has *flown under the rad*ar for many years. That said, investigators must approach this topic with a grasp of the scope of what has been done, what is known, and what needs to be addressed. This review was written with that intent.

The evidence clearly indicates that exercise training can result in the development of low testosterone in men, and at times the level of reductions reaches the clinical definition of hypogonadism. That said, some researchers support the use of terminology noting the existence of an exercise relative hypogonadism. The vast majority of the publishing findings, however, suggest the testosterone reductions found with training are in the normal clinical range (healthy, non-obese men), but frequently at the low end of the range.

**Figure 5:**
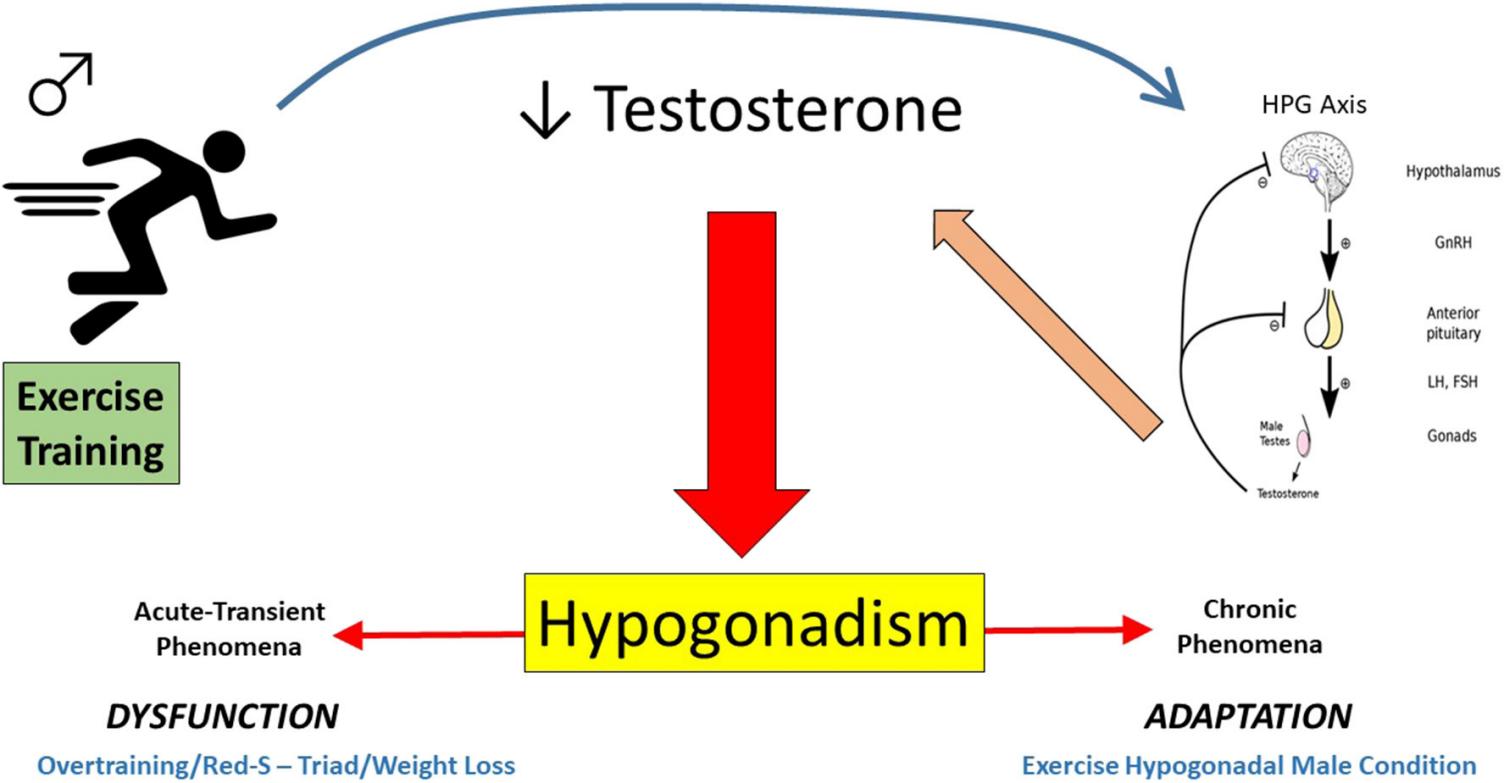
Pictorial depiction of the proposed continuum of exercise-related hypogonadism-low testosterone in exercising men (Acute-Transient = affect lasting days/weeks/months, whereas Chronic = more persistent affect displayed for years). This excludes trauma-related or anabolic androgenic steroid induced hypogonadism.

It is proposed herein, that the development of exercise relative hypogonadism from training can be generalized into one of two categories; an acute, transient phenomenon (overtraining, Triad/RED-S … *etcetera*) or a more chronic phenomenon reflective of a training-induced adaptation (EHMC). Figure 5 presents a schematic representation of the conceptual framework for the forms of exercise relative hypogonadism proposed, unrelated to trauma events or AAS use.

The physiological mechanisms by which low testosterone-hypogonadism develops presently unresolved, but theories revolve around either peripheral or central disruption of the HPG axis resulting in hypogonadotropic hypogonadism. Specifically involving either stress hormone interference or caloric deficient/energy availability compromise of the axis function. Most current contemporary research work has focused on the latter, and almost explicitly on the role of LEA associated axis disruption. Although it is important to remember that low testosterone-hypogonadism can exist in athletes-exercisers due to other scenarios such as TBI events or AAS use, and should always be ruled-out before assuming other causalities.

In looking to the future, it is important to recognize the available research literature is limited in number and need for expansion. Also, there is a need to have more replication of existing findings. Furthermore, many of the existing studies are of a retrospective, cross-sectional approach and involve small sample sizes. These types of studies are informative but more prospective, experimental research designed is needed where variables are manipulated which allows addressing of cause and effect issues. Granted, such approaches are desirable in executing the scientific method, but problematic in logistics, ethics and demanding financially. Nonetheless, they are needed.

Clinical attention is sorely needed for the male athlete-exerciser suffering from the debilitating aspects of the Overtraining Syndrome and, or Triad/RED-S conditions. First and foremost they should be the ones to be aided by future research endeavors as their health, and in some cases, livelihood is being adversely affected by their conditions. Furthermore, these individuals may suffer long-term, delayed health consequences we are currently unaware of; future researchers should examine this issue too. As to the EHMC individuals who displayed an exercise relative hypogonadism (proposed due to an adjustment in the HPG regulatory axis; i.e., allowing for a new set-point lowering of testosterone levels), it is entirely unclear is a clinical intervention is warranted (or desired) since negative health consequences are not reported. Nonetheless, more expansive healthcare assessments and evaluations based studies are recommended to ensure there are not some insidious consequences thus far undetected in such men.

Finally, it is recommended that exercise physiologists who study hormones and clinical endocrinologists who are interested in exercise attempt to work together more closely in a cooperative fashion on this issue—this has not always been the case in the past (133, 134). This type of collective team approach will most surely lead to a more clear and precise understanding of how exercise and the training process influence the reproductive system in women and men.

## Author Contributions

The author confirms being the sole contributor of this work and has approved it for publication.

### Conflict of Interest

The author declares that the research for this paper was conducted in the absence of any commercial or financial relations that could be construed as a potential conflict of interest.

## References

[1] World Health Organization Global Recommendations on Physical Activity for Health. Geneva: World Health Organization (2010). Available online at: http://www.who.int/dietphysicalactivity/publications/9789241599979/en (accessed November 01, 2019).

[2] Lee I-M Shiroma EJ Lobelo F Puska P Blair SN Katzmarzyk PT. Effect of physical inactivity on major non-communicable diseases worldwide: an analysis of burden of disease and life expectancy. Lancet. (2012) 380:219–29. 10.1016/S0140-6736(12)61031-9 22818936 PMC3645500

[3] Haskell WL Lee I-M Pate RR Powell KE Blair SN Franklin BA. Physical activity and public health: updated recommendation for adults from the American College of Sports Medicine and the American Heart Association. Med Sci Sports Exerc. (2007) 39:1423–34. 10.1249/mss.0b013e3180616b27 17762377

[4] World Health Organization Global Strategy on Diet, Physical Activity and Health. Available online at: https://www.who.int/dietphysicalactivity/factsheet_adults/en/ (accessed November 01, 2019).

[5] Pate RR Flynn JI Marsha D. Policies for promotion of physical activity and prevention of obesity in adolescence. J Exerc Sci Fit. (2016) 14:47–53. 10.1016/j.jesf.2016.07.003 29541118 PMC5801719

[6] Mountjoy M Sundgot-Borgen J Burke L Carter S Constantini N Lebrun C. The IOC consensus statement: beyond the Female Athlete Triad—Relative Energy Deficiency in Sport (RED-S). Br J Sports Med. (2014) 48:491–7. 10.1136/bjsports-2014-093502 24620037

[7] Nattiv A Loucks AB Manore MM Sanborn CF Sundgot-Borgen J Warren MP American College of Sports Medicine. American College of Sports Medicine position stand. The female athlete triad. Med Sci Sports Exerc. (2007) 39:1867–82. 10.1249/mss.0b013e318149f111 17909417

[8] Rey RA Grinspon RP Gottlieb S Pasqualini T Knoblovits P Aszpis S. Male hypogonadism: an extended classification based on a developmental, endocrine physiology-based approach. Andrology. (2013) 1:3–16. 10.1111/j.2047-2927.2012.00008.x 23258624

[9] Kumar P Kumar N Thakur DS Patidar A. Male hypogonadism: symptoms and treatment. J Adv Pharm Technol Res. (2010) 1:297–301. 10.4103/0110-5558.72420 22247861 PMC3255409

[10] Sterling J Bernie AM Ramasamy R. Hypogonadism: easy to define, hard to diagnose, and controversial to treat. Can Urol Assoc J. (2015) 9:65–8. 10.5489/cuaj.2416 25737761 PMC4336035

[11] Rivier C Rivest S. Effect of stress on the activity of the hypothalamic-pituitary-gonadal axis: peripheral and central mechanisms. Biol Reprod. (1991) 45:523–32. 10.1095/biolreprod45.4.523 1661182

[12] Bhasin S Brito JP Cunningham GR Hayes FJ Hodis HN Matsumoto AM. Testosterone therapy in men with hypogonadism: an Endocrine Society clinical practice guideline. J Clin Endocrinol Metab. (2018) 103:1715–44. 10.1210/jc.2018-00229 29562364

[13] Pivonello R Menafra D Riccio E Garifalos F Mazzella M de Angelis C. Metabolic disorders and male hypogonadotropic hypogonadism. Front Endocrinol. (2019) 10:345. 10.3389/fendo.2019.00345 31402895 PMC6669361

[14] Mayo Clinic Web Site. Testosterone. Available online at: https://www.mayocliniclabs.com/test-catalog/Clinical+and+Interpretive/83686 (accessed October 07, 2019).

[15] Travison TG Vesper HW Orwoll E Wu F Kaufman JM Wang Y. Harmonized reference ranges for circulating testosterone levels in men of four cohort studies in the United States and Europe. J Clin Endocrinol Metab. (2017) 102:1161–73. 10.1210/jc.2016-2935 28324103 PMC5460736

[16] Handelsman DJ Hirschber AL Bermon S. Circulating testosterone as the hormonal basis of sex differences in athletic performance. Endocrine Rev. (2018) 39:803–29. 10.1210/er.2018-00020 30010735 PMC6391653

[17] Camporesi S. When does an advantage become unfair? Empirical and normative concerns in Semenya's case. J Med Ethics. (2019) 45:700–4. 10.1136/medethics-2019-105532 31527141

[18] Aversa A Morgentaler A. The practical management of testosterone deficiency in men. Nat Rev Urol. (2015) 12:641–50. 10.1038/nrurol.2015.238 26458755

[19] Hackney AC Moore AW Brownlee KK. Testosterone and endurance exercise: development of the exercise-hypogonadal male condition. Acta Physiol. (2005) 92:121–37. 10.1556/APhysiol.92.2005.2.3 16268050

[20] Hackney AC Fahrner CL Gulledge TP. Basal reproductive hormonal profiles are altered in endurance trained men. J Sports Med Phys Fit. (1998) 38:138–41. 9763799

[21] Hooper DR Tenforde AS Hackney AC. Treating exercise-associated low testosterone and its related symptoms. Phys Sportsmed. (2018) 46:427–34 10.1080/00913847.2018.1507234 30063407

[22] Jürimäe J Mäestu J Purge P Jürimäe T. Changes in stress and recovery after heavy training in rowers. J Sci Med Sport. (2004) 7:335–9. 10.1016/S1440-2440(04)80028-8 15518298

[23] Ayers JW Komesu Y Romani T Ansbacher R. Anthropomorphic, hormonal, and psychologic correlates of semen quality in endurance-trained male athletes. Fertil Steril. (1985) 43:917–21. 10.1016/S0015-0282(16)48622-X 3158553

[24] Hackney AC Lane AR Increased prevalence of androgen deficiency in endurance-trained male runners across the life span. Aging Male. (2018) 20:1 10.1080/13685538.2018.1523888 30457033

[25] Hackney AC Hooper DR. Low testosterone—androgen deficiency, endurance exercise training and competitive performance. Physiol Int. (2019) 106:379–89. 10.1556/2060.106.2019.30 31847532

[26] Hackney AC Hackney ZC. The exercise hypogonadal male condition and endurance exercise training. Curr Trends Endocrinol. (2005) 1:101–6. 31723314 PMC6853631

[27] Hsu B Cumming RG Handelsman DJ. Testosterone, frailty and physical function in older men. Exp Rev Endocrinol Metab. (2018) 13:159–65. 10.1080/17446651.2018.1475227 30058896

[28] Decaroli MC Rochira V. Aging and sex hormones in males. Virulence. (2017) 8:545–70. 10.1080/21505594.2016.1259053 27831823 PMC5538340

[29] Rooyackers OE Nair KS. Hormonal regulation of human muscle protein metabolism. Ann Rev Nutr. (1997) 17:457–85. 10.1146/annurev.nutr.17.1.457 9240936

[30] Sinha-Hikim I Artaza J Woodhouse L Gonzalez-Cadavid N Singh AB Lee MI. Testosterone-induced increase in muscle size in healthy young men is associated with muscle fiber hypertrophy. Am J Physiol. (2002) 283:E154–64. 10.1152/ajpendo.00502.2001 12067856

[31] West D Phillips S. Anabolic processes in human skeletal muscle: restoring the identities of growth hormone and testosterone. Phys Sportsmed. (2010) 38:97–104. 10.3810/psm.2010.10.1814 20959702

[32] Bhasin S Woodhouse L Casaburi R Singh AB Bhasin D Berman N. Testosterone dose-response relationships in healthy young men. Am J Physiol. (2001) 281:E1172–81. 10.1152/ajpendo.2001.281.6.E1172 11701431

[33] Hooper DR Kraemer WJ Focht BC Volek JS DuPont WH Caldwell LK. Endocrinological roles for testosterone in resistance exercise responses and adaptations. Sports Med. (2017) 47:1709–20. 10.1007/s40279-017-0698-y 28224307

[34] Staerman F Léon P. Andropause (androgen deficiency of the aging male): diagnosis and management. Minerva Medica. (2012) 103:333−42. 23042368

[35] Hackney AC Lane AR. Low testosterone in male endurance-trained distance runners: impact of years in training. Hormones. (2018) 17:137–9. 10.1007/s42000-018-0010-z 29858867

[36] Rigotti NA Neer RM Jameson L. Osteopenia and bone fractures in a man with anorexia nervosa and hypogonadism. JAMA. (1986) 256:385–8. 10.1001/jama.1986.03380030087034 3723727

[37] Arce JC De Souza MJ Pescatello LS Luciano AA. Subclinical alterations in hormone and semen profile in athletes. Fertil Steril. (1993) 59:398–404. 10.1016/S0015-0282(16)55684-2 8425638

[38] Bennell KL Brukner PD Malcolm SA. Effect of altered reproductive function and lowered testosterone levels on bone density in male endurance athletes. Br J Sports Med. (1996) 30:205–8. 10.1136/bjsm.30.3.205 8889111 PMC1332330

[39] McBride JA Carson CC Coward RM. Testosterone deficiency in the aging male. Therapeut Adv Urol. (2016) 8:47–60. 10.1177/1756287215612961 26834840 PMC4707424

[40] Sutton JR Coleman MJ Casey J Lazarus L. Androgen responses during physical exercise. Br Med J. (1973) 1:520–2. 10.1136/bmj.1.5852.520 4692677 PMC1588661

[41] Wheeler GD Wall SR Belcastro AN Cumming DC Reduced serum testosterone and prolactin levels in male distance runners. JAMA. (1984) 27:514–6. 10.1001/jama.1984.03350040044020 6429357

[42] Hackney AC Dolny DG Ness RJ Comparison of reproductive hormonal profiles in select athletic groups. Biol Sport. (1988) 5:297–304.

[43] Hackney AC Sinning WE Brout BC Comparison of resting reproductive hormonal profiles in endurance trained and untrained men. Med Sci Sports Exerc. (1988) 20:60–5. 10.1249/00005768-198802000-00009 3343919

[44] Wheeler GD Wall SR Belcastro AN Conger P Cumming DC. Are anorexic tendencies prevalent in the habitual runner? Br J Sports Med. (1986) 20:77–81. 10.1136/bjsm.20.2.77 3488092 PMC1478310

[45] MacConnie SE Barkan A Lampman RM Schork MA Beitins IZ. Decreased hypothalamic gonadotropin-releasing hormone secretion in male marathon runners. N Engl J Med. (1986) 315:411–7. 10.1056/NEJM198608143150702 3090437

[46] Fink J Schoenfeld BJ Nakazato K. The role of hormones in muscle hypertrophy. Phys Sportsmed. (2018) 46:129–34. 10.1080/00913847.2018.1406778 29172848

[47] Kraemer RR Kilgore JL Kraemer GR Castracane VD. Growth hormone, IGF-I, and testosterone responses to resistive exercise. Med Sci Sports Exerc. (1992) 24:1346–52. 10.1249/00005768-199212000-00007 1470017

[48] Vingren JL Kraemer WJ Ratamess NA Anderson JM Volek JS Maresh CM. Testosterone physiology in resistance exercise and training: the up-stream regulatory elements. Sports Med. (2010) 40:1037–53. 10.2165/11536910-000000000-00000 21058750

[49] Viru A Hormones in Muscular Activity. Boca Raton, FL: CRC Press Inc (1985).

[50] Hackney AC. Diurnal hormonal responses in exercise and sports medicine research: range effect adjustments. Biomed Hum Kinet. (2010) 2:85–8. 10.2478/v10101-0021-y 29973983 PMC6028039

[51] Hackney AC Lane AR. Exercise and the regulation of endocrine hormones. Progr Mol Biol Transl Sci. (2015) 135:293–311. 10.1016/bs.pmbts.2015.07.001 26477919

[52] Boer K Exercise Endocrinology. Champaign, IL: Human Kinetics Publishing (2003).

[53] Lima-Oliveira G Volanski W Lippi G Picheth G Guidi GC. Pre-analytical phase management: a review of the procedures from patient preparation to laboratory analysis. Scand J Clin Lab Inv. (2017) 77:153–63. 10.1080/00365513.2017.1295317 28266238

[54] Adebero T McKinlay BJ Theocharidis A Root Z Josse AR Klentrou P Salivary and serum concentrations of cortisol and testosterone at rest and in response to intense exercise in boys versus men. Pediatr Exerc Sci. (2019) 25:1–8. 10.1123/pes.2019-0091 31770720

[55] VanBruggen MD Hackney AC McMurray RG Ondrak KS. The relationship between serum and salivary cortisol levels in response to different intensities of exercise. Int J Sports Physiol Perform. (2011) 6:396–407. 10.1123/ijspp.6.3.396 21911864

[56] Kuipers H Keizer HA. Overtraining in elite athletes. Review and directions for the future. Sports Med. (1988) 6:79–92. 10.2165/00007256-198806020-00003 3062735

[57] Budgett R Newsholme E Lehmann M Sharp C Jones D Peto T. Redefining the overtraining syndrome as the unexplained underperformance syndrome. Br J Sports Med. (2000) 34:67–8. 10.1136/bjsm.34.1.67 10690455 PMC1724136

[58] Cadegiani FA Kater CE. Novel insights of overtraining syndrome discovered from the EROS study. BMJ Open. (2019) 5:e000542. 10.1136/bmjsem-2019-000542 31297238 PMC6590962

[59] Cadegiani FA Kater CE. Basal hormones and biochemical markers as predictors of overtraining syndrome in male athletes: the EROS-BASAL study. J Athl Train. (2019) 54:906–14. 10.4085/1062-6050-148-18 31386577 PMC6756603

[60] Jezler A Sportaerztliche Aufgaben. Schweizer Medizinische Wochenschrift. (1939) 7:151–155.

[61] Armstrong LE VanHeest JL. The unknown mechanism of the Overtraining Syndrome. Sports Med. (2002) 32:185–209. 10.2165/00007256-200232030-00003 11839081

[62] Aaakvaag A Sand T Opstad PK Fonnum F Hormonal changes in serum in young men during prolonged physical strain. Eur J Appl Physiol. (1978) 39:283–91. 10.1007/BF00421452 710393

[63] Adlercreutz H Hlrkdnen M Kuoppasalmi K Kosunen K Näveri H Rehunen S. Physical activity and hormones. Adv Cardiol. (1976) 18:144–57. 10.1159/000399520 983845

[64] Dessypris A Adlercreutz H Serum total/free testosterone and sex hormone binding globulin binding capacity (SHBG) in a noncompetitive marathon run Acta Endocrinologica. (1984) 265:18–9. 10.1530/acta.0.107S0018 6593980

[65] Harkönen M Kuoppasalmi K Näveri H Tikkanen H Icén A Adlercreutz H Biochemical indicators in diagnosis of overstrain condition in athletes. In: Sports Medicine and Exercise Science, Proceedings of the Olympic Scientific Congress. Eugene, OR (1984).

[66] Remes K Kuoppasalmi K Adlercreutz H Effect of long term physical exercise training on plasma testosterone, androstenedione, luteinizing hormone and sex-hormone-binding globulin capacity. Scand J Clin Lab Invest. (1979) 39:743–9. 10.1080/00365517909108166 575229

[67] Seene T Viru A The catabolic effect of glucocorticoids on different types of skeletal muscle fibers and its dependence upon muscle activity and interaction with anabolic steroids. J Steroid Biochem. (1982) 16:349–52. 10.1016/0022-4731(82)90190-X 7043094

[68] Adlercreutz H Härkönen M Kuoppasalmi K Nãveri H Huhtaniemi I Tikkanen H. Effect of training on plasma anabolic and catabolic steroid hormones and their response during physical exercise. Int J Sports Med. (1986) 7:27–8. 10.1055/s-2008-1025798 3744643

[69] Dressendorfer RH Wade CE Iverson D Decreased plasma testosterone in overtrained runners. Med Sci Sports Exerc. (1987) 19(Suppl.):10 10.1249/00005768-198704001-00058

[70] Griffith RB Dressendorfer RH Fullbright CD Effects of over-work on testosterone, sperm count and libido. Med Sci Sports Exerc. (1988) 20(Suppl.):39

[71] Fry RW Morton AR, Garcia-Webb P. Biological responses to overload training in endurance sports. Eur J Appl Physiol. (1992) 64:335–44. 10.1007/BF00636221 1592059

[72] Roberts AC McClure RD Weiner RI Brooks GA. Overtraining affects male reproductive status. Fertil Steril. (1993) 60:686–92. 10.1016/S0015-0282(16)56223-2 8405526

[73] Hackney AC Hormonal changes at rest in overtrained endurance athletes. Biol Sport. (1991) 8:49–56. PMC7098450 32218643

[74] Urhausen A Gabriel H Kindermann W. Urhausen A Gabriel H Kindermann W. Blood hormones as markers of training stress and overtraining. Sports Med. (1995) 20:251–76. 10.2165/00007256-199520040-00004 8584849

[75] Kreher JB Schwartz JB. Overtraining syndrome: a practical guide. Sports Health. (2012) 4:128–38. 10.1177/1941738111434406 23016079 PMC3435910

[76] Lee EC Fragala MS Kavouras SA Queen RM Pryor JL Casa DJ. Biomarkers in sports and exercise: tracking health, performance, and recovery in athletes. J Strength Condition Res. (2017) 31:2920–37. 10.1519/JSC.0000000000002122 28737585 PMC5640004

[77] Meeusen R Duclos M Foster C Fry A Gleeson M Nieman D. Prevention, diagnosis, and treatment of the overtraining syndrome: joint consensus statement of the European College of Sports Science and the American College of Sports Medicine. Med Sci Sports Exerc. (2013) 45:186–205. 10.1249/MSS.0b013e318279a10a 23247672

[78] Doerr P Pirke KM. Cortisol-induced suppression of plasma testosterone in normal adult males. J Clin Endocrinol Metab. (1976) 43:622–9. 10.1210/jcem-43-3-622 956348

[79] Cumming DC Quigley ME Yen SS. Acute suppression of circulating testosterone levels by cortisol in men. J Clin Endocrinol Metab. (1983) 57:671–3. 10.1210/jcem-57-3-671 6348068

[80] Anderson T Lane AR Hackney AC. Cortisol and testosterone dynamics following exhaustive endurance exercise. Eur J Appl Physiol. (2016) 116:1503–9. 10.1007/s00421-016-3406-y 27262888

[81] Urhausen A Kullmer T Kindermann W. A 7-week follow-up study of the behaviour of testosterone and cortisol during the competition period in rowers. Eur J Appl Physiol. (1987) 56:528–33. 10.1007/BF00635365 3653093

[82] Brownlee KK Moore AW Hackney AC. Relationship between circulating cortisol and testosterone: influence of physical exercise. J Sports Sci Med. (2005) 4:76–83. 24431964 PMC3880087

[83] Narayan E Parisella S Influence of the stress endocrine system on the reproductive endocrine axis in sheep (*Ovis aries*). Ital J Anim Sci. (2017) 4:640–51. 10.1080/1828051X.2017.1321972

[84] Hackney AC Saeidi A. The thyroid axis, prolactin, and exercise in humans. Curr Opin Endoc Metab Res. (2019) 9:45–50. 10.1016/j.coemr.2019.06.012 31482146 PMC6720127

[85] Tovar S Diéguez C. Prolactin and energy homeostasis: pathophysiological mechanisms and therapeutic considerations. Endocrinology. (2014) 155:659–62. 10.1210/en.2013-2167 24564416

[86] Ben-Jonathan N Hugo ER Brandebourg TG LaPensee CR. Focus on prolactin as a metabolic hormone. Trends Endocrinol. (2006) 17:110–6. 10.1016/j.tem.2006.02.005 16517173

[87] Hackney AC Fahrner CL Stupnicki R. Reproductive hormonal responses to maximal exercise in endurance-trained men with low resting testosterone levels. Exp Clin Endocrinol Diab. (1997) 105:291–5. 10.1055/s-0029-1211767 9354858

[88] Hackney AC Sharp RL Runyan WS Ness RJ. Relationship of resting prolactin and testosterone in males during intensive training. Br J Sports Med. (1989) 23:194. 10.1136/bjsm.23.3.194 19650255 PMC1478682

[89] Wheeler GD Singh M Pierce WD Epling WF Cumming DC. Endurance training decreases serum testosterone levels in men without change in luteinizing hormone pulsatile release. J Clin Endocrinol Metab. (1991) 72:422–5. 10.1210/jcem-72-2-422 1899423

[90] Viru A Viru M. Cortisol–essential adaptation hormone in exercise. Int J Sports Med. (2004) 25:461–4. 10.1055/s-2004-821068 15346236

[91] Anderson T Lane AR Hackney AC. The cortisol awakening response: association with training load in endurance Runners. Int J Sports Physiol Perform. (2018) 13:1158–63. 10.1123/ijspp.2017-0740 29584528

[92] Kilbanski A Beitins IZ Badger T Kittle R McArthur JW Reproductive function during fasting in men. J Clin Endocrinol Metab. (1981) 53:258–63. 10.1210/jcem-53-2-258 6788791

[93] Röjdmark S. Influence of short-term fasting on the pituitary-testicular axis in normal men. Hormone Res. (1987) 25:140–6. 10.1159/000180645 3106181

[94] Cangemi R Friedmann AJ Holloszy JO Fontana L. Long-term effects of calorie restriction on serum sex hormone concentrations in men. Aging Cell. (2010) 9:236–42. 10.1111/j.1474-9726.2010.00553.x 20096034 PMC3569090

[95] Bergendahl M Perheentupa A Huhtaniemi I. Starvation-induced suppression of pituitary-testicular function in rats is reversed by pulsatile gonadotropin-releasing hormone substitution. Biol Reprod. (1991) 44:413–9. 10.1095/biolreprod44.3.413 1901739

[96] Wong HK Hoermann R Grossmann M. Reversible male hypogonadotropic hypogonadism due to energy deficit. Clin Endocrinol. (2019) 91:3–9. 10.1111/cen.13973 30903626

[97] Dwyer AA Chavan NR Lewkowitz-Shpuntoff H Plummer L Hayes FJ Seminara SB. Functional hypogonadotropic hypogonadism in men: underlying neuroendocrine mechanisms and natural history. J Clin Endocrinol Metab. (2019) 104:3403–14. 10.1210/jc.2018-02697 31220265 PMC6594303

[98] Shimon I Lubina A Gorfine M Ilany J. Feedback inhibition of gonadotropins by testosterone in men with hypogonadotropic hypogonadism: comparison to the intact pituitary-testicular axis in primary hypogonadism. J Androl. (2006) 27:358–64. 10.2164/jandrol.05140 16474013

[99] Strauss RH Lanese RR Malarkey WB. Weight loss in amateur wrestlers and its effect on serum testosterone levels. JAMA. (1985) 254:3337–8. 10.1001/jama.254.23.3337 4068168

[100] Irfan Y. Associations among dehydration, testosterone and stress hormones in terms of body weight loss before competition. Am J Med Sci. (2015) 350:103–8. 10.1097/MAJ.0000000000000521 26125543

[101] Hackney A C Sinning WE The effects of wrestling training on reproductive hormones. Med Sci Sport Exerc. (1986) 18(Suppl.):S40 10.1249/00005768-198604001-00197

[102] Abedelmalek S Chtourou H Souissi N Tabka Z. Caloric restriction effect on proinflammatory cytokines, growth hormone, and steroid hormone concentrations during exercise in Judokas. Oxid Med Cell Longev. (2015) 2015:809492. 10.1155/2015/809492 26075039 PMC4446567

[103] Rich PA Villani R Fulton A Ashton J Bass S Brinkert R. Serum cortisol concentration and testosterone to cortisol ratio in elite prepubescent male gymnasts during training. Eur J Appl Physiol. (1992) 65:399–402. 10.1007/BF00243504 1425643

[104] Callister R Callister RJ Fleck SJ. Physiological and performance responses to overtraining in elite judo athletes. Med Sci Sports Exerc. (1990) 22:816–24. 10.1249/00005768-199012000-00014 2287260

[105] Roemmich JN Sinning WE. Weight loss and wrestling training: effects on growth-related hormones. J Appl Physiol. (1997) 82:1760–4. 10.1152/jappl.1997.82.6.1760 9173938

[106] Hobbs JG Young JS Bailes JE. Sports-related concussions: diagnosis, complications, and current management strategies. Neurosurgical Focus. (2016) 40:E5. 10.3171/2016.1.FOCUS15617 27032922

[107] Scranton RA Baskin DS. Impaired pituitary axes following traumatic brain injury. J Clin Med. (2015) 4:1463–79. 10.3390/jcm4071463 26239686 PMC4519800

[108] Grashow R Weisskopf MG Miller KK Nathan DM Zafonte R Speizer FE Association of concussion symptoms with testosterone levels and erectile dysfunction in former professional us-style football players. JAMA Neurol. (2019) 26:e192664 10.1001/jamaneurol.2019.2664 PMC6714010 31449296

[109] Loucks AB Kiens B Wright HH. Energy availability in athletes. J Sports Sci. (2011) 29(Suppl. 1):S7–15. 10.1080/02640414.2011.588958 21793767

[110] DeSouza MJ Koltun KJ Williams NI The role of energy availability in reproductive function in the female athlete triad and extension of its effects to men: an initial working model of a similar syndrome in male athletes. Sports Med. (2019) 49(Suppl. 2):125–37. 10.1007/s40279-019-01217-3 31696452 PMC6901401

[111] Cumming DC Wheeler GD McColl EM. The effects of exercise on reproductive function in men. Sports Med. (1989) 7:1–17. 10.2165/00007256-198907010-00001 2652242

[112] Hackney AC. Endurance training and testosterone levels. Sports Med. (1989) 8:117–27. 10.2165/00007256-198908020-00004 2675257

[113] Mountjoy M Sundgot-Borgen J Burke L Ackerman KE Blauwet C Constantini N. International Olympic Committee (IOC) Consensus statement on Relative Energy Deficiency in Sport (RED-S): 2018 update. Int J Sport Nutr Exerc Metab. (2018) 28:316–31. 10.1123/ijsnem.2018-0136 29771168

[114] Marcason W. Female athlete triad or relative energy deficiency in sports (red-s): is there a difference? J Acad Nutr Diet. (2016) 116:744. 10.1016/j.jand.2016.01.021 27017180

[115] Hooper DR Kraemer WJ Saenz C Schill KE Focht BC Volek JS. The presence of symptoms of testosterone deficiency in the exercise-hypogonadal male condition and the role of nutrition. Eur J Appl Physiol. (2017) 117:1349–57. 10.1007/s00421-017-3623-z 28470410

[116] Hooper DR Kraemer WJ Stearns RL Kupchak BR Volk BM DuPont WH. Evidence of the exercise hypogonadal male condition at the 2011 Kona Ironman World Championships. Int J Sports Physiol Perform. (2018) 14:170–5. 10.1123/ijspp.2017-0476 29952670

[117] Elliott-Sale KJ Tenforde AS Parziale AL Holtzman B Ackerman KE. Endocrine effects of relative energy deficiency in sport. Int J Sport Nutr Exerc Metab. (2018) 28:335–49. 10.1123/ijsnem.2018-0127 30008240

[118] Pope HG Jr Wood RI Rogol A Nyberg F Bowers L Bhasin S. Adverse health consequences of performance-enhancing drugs: an Endocrine Society scientific statement. Endocrine Rev. (2014) 35:341–75. 10.1210/er.2013-1058 24423981 PMC4026349

[119] Hackney AC. Effects of endurance exercise on the reproductive system of men: the exercise-hypogonadal male condition. J Endocrinol Invest. (2008) 31:932–8. 10.1007/BF03346444 19092301

[120] Hew-Butler T Jordaan E Noakes TD Soldin SJ Verbalis JG Hypogonadal male runners do not display endocrine or performance decrements during prolonged endurance exercise. FASEB J. (2009) 23(Suppl. 1):955.14.

[121] Pontzer H. Energy constraint as a novel mechanism linking exercise and health. Physiology. (2018) 33:384–93. 10.1152/physiol.00027.2018 30303776

[122] Raichlen DA Pontzer H Harris JA Mabulla AZ Marlowe FW Josh Snodgrass J. Physical activity patterns and biomarkers of cardiovascular disease risk in hunter-gatherers. Am J Hum Biol. (2017) 29:e22919. 10.1002/ajhb.22919 27723159

[123] Trumble BC Cummings D von Rueden C O'Connor KA Smith EA Gurven M. Physical competition increases testosterone among Amazonian forager-horticulturalists: a test of the 'challenge hypothesis'. Proc R Soc. (2012) 279:2907–12. 10.1098/rspb.2012.0455 22456888 PMC3367794

[124] Ellison PT Bribiescas RG Bentley GR Campbell BC Lipson SF Panter-Brick C. Population variation in age-related decline in male salivary testosterone. Hum Reprod. (2002) 17:3251–3. 10.1093/humrep/17.12.3251 12456632

[125] Hackney AC Sinning WE Bruot BC. Hypothalamic-pituitary-testicular axis function in endurance-trained males. Int J Sports Med. (1990) 11:298–303. 10.1055/s-2007-1024811 2228360

[126] Hackney AC Szczepanowska E Viru AM. Basal testicular testosterone production in endurance-trained men is suppressed. Eur J Appl Physiol. (2003) 89:198–201. 10.1007/s00421-003-0794-6 12665985

[127] Bobbert T Brechtel L Mai K Otto B Maser-Gluth C Pfeiffer AF. Adaptation of the hypothalamic-pituitary hormones during intensive endurance training. Clin Endocrinol. (2005) 63:530–6. 10.1111/j.1365-2265.2005.02377.x 16268805

[128] Sansone A Sansone M Vaamonde D Sgrò P Salzano C Romanelli F. Sport, doping and male fertility. Reprod Biol Endocrinol. (2018) 16:114. 10.1186/s12958-018-0435-x 30415644 PMC6231265

[129] World Anti Doping Agency Prohibited List Available online at: https://www.wada-ama.org/en/prohibited-list (accessed September 20, 2019).

[130] Topo E Soricelli A D'Aniello A Ronsini S D'Aniello G. The role and molecular mechanism of D-aspartic acid in the release and synthesis of LH and testosterone in humans and rats. Reprod Biol Endocrinol. (2009) 7:120. 10.1186/1477-7827-7-120 19860889 PMC2774316

[131] Rao A Steels E Inder WJ Abraham S Vitetta L. Testofen, a specialised Trigonella foenum-graecum seed extract reduces age-related symptoms of androgen decrease, increases testosterone levels and improves sexual function in healthy aging males in a double-blind randomised clinical study. Aging Male. (2016) 19:134–42. 10.3109/13685538.2015.1135323 26791805

[132] Hackney AC Athlete testing, analytical procedures, and adverse analytical findings. 1st ed In: Doping, Performance Enhancing-Drug, and Hormones in Sport. New York, NY: Elsevier Publishing (2018). p. 113–28. 10.1016/B978-0-12-813442-9.00010-9

[133] Hackney AC Viru A. Research methodology: endocrinologic measurements in exercise science and sports medicine. J Athl Train. (2008) 43:631–9. 10.4085/1062-6050-43.6.631 19030142 PMC2582556

[134] Di Luigi L Romanelli F Sgrò P Lenzi A. Andrological aspects of physical exercise and sport medicine. Endocrine. (2012) 42:278–84. 10.1007/s12020-012-9655-6 22430368

